# Hemifacial Spasm and Neurovascular Compression

**DOI:** 10.1155/2014/349319

**Published:** 2014-10-28

**Authors:** Alex Y. Lu, Jacky T. Yeung, Jason L. Gerrard, Elias M. Michaelides, Raymond F. Sekula, Ketan R. Bulsara

**Affiliations:** ^1^Department of Neurosurgery, Yale School of Medicine, New Haven, CT 06520, USA; ^2^Section of Otolaryngology, Yale School of Medicine, New Haven, CT 06520, USA; ^3^Hamot Hospital, University of Pittsburgh Medical Center, Erie, PA 16507, USA

## Abstract

Hemifacial spasm (HFS) is characterized by involuntary unilateral contractions of the muscles innervated by the ipsilateral facial nerve, usually starting around the eyes before progressing inferiorly to the cheek, mouth, and neck. Its prevalence is 9.8 per 100,000 persons with an average age of onset of 44 years. The accepted pathophysiology of HFS suggests that it is a disease process of the nerve root entry zone of the facial nerve. HFS can be divided into two types: primary and secondary. Primary HFS is triggered by vascular compression whereas secondary HFS comprises all other causes of facial nerve damage. Clinical examination and imaging modalities such as electromyography (EMG) and magnetic resonance imaging (MRI) are useful to differentiate HFS from other facial movement disorders and for intraoperative planning. The standard medical management for HFS is botulinum neurotoxin (BoNT) injections, which provides low-risk but limited symptomatic relief. The only curative treatment for HFS is microvascular decompression (MVD), a surgical intervention that provides lasting symptomatic relief by reducing compression of the facial nerve root. With a low rate of complications such as hearing loss, MVD remains the treatment of choice for HFS patients as intraoperative technique and monitoring continue to improve.

## 1. Clinical Features

HFS starts with tonic-clonic contractions of the orbicularis oculi muscle, resulting in involuntary eyelid closure and eyebrow elevation. Over time, the contractions progress to the region affecting the frontalis (i.e., muscles of the forehead), platysma (i.e., muscles of the neck), and orbicularis oris (i.e., muscles of the mouth) muscles [1–5]. Eventually, the patient may develop sustained contractions of all involved muscles, causing a severe, disfiguring grimace with partial closure of the eyes and lifting of the mouth corners in the “tonus phenomenon” [3]. The majority of HFS cases occur unilaterally with an estimated 0.6% to 5% occurring bilaterally [6].

Some patients will report worsening of spasms with fatigue, situations of anxiety, and changes in position of the head (e.g., head to one side or the other on the pillow at night) [7]. One study also found that HFS-related headaches were associated with increased spasm severity [8]. Another study suggested that HFS patients have a higher chance than the general American population (15.1% versus 1.34%, *P* < 0.001) of presenting with rosacea, a chronic condition characterized by facial erythema, fine telangiectasia, papules ocular irritation, and rhinophyma [9].

## 2. Epidemiology

HFS is prevalent in 9.8 per 100,000 persons [10]. The average age of onset for HFS is 44 years. Women and Asian populations have an increased susceptibility to HFS though valid prevalence data is scarce [11–13]. This issue is due to HFS underdiagnosis, misdiagnosis, and absence of population-based data [14]. A study of 203 family physicians in 2004 found that 90.6% were unable to diagnose HFS correctly and that 46.3% did not know how to manage HFS [15]. Worldwide estimates for the prevalence of HFS are 14.5 per 100,000 women and 7.4 per 100,000 men [16, 17].

Families with HFS present with autosomal dominant inheritance and low penetrance although there have been only a few reported cases [18]. In addition, the genetic susceptibility is poorly defined as there is not a clear relationship between HFS and single-nucleotide polymorphisms in genes related to vascular compression [19].

## 3. Pathophysiology

The accepted underlying pathophysiology of HFS suggests that the disease process is caused by facial nerve root entry zone myelin breakdown and ephaptic transmission, which is the passage of neural impulses through artificial chemical or chemical synapses. The root exit zone of the facial nerve is defined as the transition point between central (oligodendrocytes) and peripheral (Schwann) cell myelination [20, 21]. This segment is sheathed by only an arachnoidal membrane and lacks both interfascicular connective tissue separating fibers and epineurium; these features increase this segment's vulnerability to compression [21]. Compared to similar disorders of the trigeminal, glossopharyngeal, and vagus nerves, a study correlated the length and volume of central myelin portions of these nerves with the incidence of the nerves' corresponding diseases [22]. One study suggested that the root exit zone was primarily involved in only 23% of its studied HFS patients whereas compression of a more proximal segment of the facial nerve when it emerges from the pontomedullary sulcus was implicated in 73% [23].

Ectopic excitation can result from an area along the nerve that generates impulses independently of the natural synapse when the excitation threshold is low due to processes such as demyelination. One study examined orbicularis oris muscle response using EMG after supraorbital nerve stimulation and lateral spread tests with diazepam injections; the study results showed consistent latent muscle responses, which implicate ectopic excitation and ephaptic transmission [24].

Two hypotheses for the hypotheses of the HFS pathophysiology exist. The nuclear/central hypothesis suggests that injury to the facial nerve causes regressive medullary changes with functional connective reorganization in the facial nucleus, causing nuclear hyperexcitability because of dendritic spike generation [20]. The peripheral hypothesis suggests that clinical symptoms result from ectopic impulse generation and “cross-talk” between fibers at site of the lesions [20]. However, these hypotheses fall short with abnormal muscle response (AMR) data. When using electrophysiological monitoring stimulating one branch of the facial nerve while recording from muscles innervated by other branches of the facial nerve, HFS patients generate a characteristic wave with a latency of approximately 10 milliseconds, which is defined as the AMR [25, 26]. Theoretically, the latency of the AMR should equal the sum of the latency of the stimulus delivered to the facial nerve branch and recorded at the vascular compression site as well as the latency from direct facial root stimulation at the site of vascular compression and the resulting muscle depolarization [27]. However, the sum of these latencies is 2 milliseconds less than the expected total [28], which cannot be explained by the central or peripheral hypotheses.

Another hypothesis is the sympathetic hypothesis. The adventitia of arteries contains sympathetic endings and is worn down in HFS, causing neurotransmitters to induce ectopic action potentials that travel to the neuromuscular junction and induce involuntary contraction of facial muscles [27]. Using HFS rat models and electrophysiological monitoring, neurotransmitter released from autonomic nervous endings in the adventitia of offending vessels induced ectopic action potentiation in demyelinated facial nerve fibers [29].

## 4. Etiology 

The etiology of HFS can be divided into two types: primary and secondary. Primary HFS is defined by vascular compression of the facial nerve root entry zone in the posterior fossa [30, 31]. Implicated arteries include the anterior inferior cerebellar artery (AICA), posterior inferior cerebellar artery (PICA), and vertebral artery (VA). Anatomic variations in vasculature such as lateral deviation of one or both vertebral arteries occurred on the ipsilateral side of HFS in 86.4% cases, making these variations a HFS risk factor [32]. The pattern of neurovascular compression can be divided into six different categories: (A) loop type, where the vascular itself creates the compression, (B) arachnoid type, where arachnoid trabeculae between the vessel and brainstem cause the vessel to tether to the nerve, (C) perforator type, where the perforating arteries from the compressing vessel tether the vessel to the brainstem, (D) branch type, where the nerve is caught between the compressing vessel and its branches, (E) sandwich type, where the nerve is sandwiched between two different vessels, and (F) tandem type, where one vessel compresses another vessel that compresses the nerve [33]. Multiple vessel compressions have been observed in 38% of HFS cases [23]. However, many patients present without an identifiable etiology [34]. Some studies have shown a higher prevalence of hypertension in patients with primary HFS compared to patients with other neurological diseases [1, 35, 36]. The association suggests that hypertension leads to arterial vessel ectasia and contributes to neurovascular compression of the facial nerve [1].

Secondary HFS occurs with damage anywhere along the facial nerve from the internal auditory canal to the stylomastoid foramen [30]. Cases of secondary HFS have been linked to cerebellopontine angle (CPA) tumors and vascular malformations with other case linked to facial nerve trauma, demyelinating lesions, and vascular insults [34]. CPA tumors occur rarely; in a study of 2,050 HFS cases, only nine patients had HFS that was attributable to CPA tumors, which included two vestibular schwannomas, five meningiomas, and two epidermoid tumors [37]. Mechanisms of HFS in this study also differed with six cases identifying offending vessels as well as individual cases of tumor encasement of the facial nerve, hypervascular tumor compression of the facial nerve, and a large tumor compressing the brain stem causing contralateral facial nerve compression [37]. Young onset HFS has been linked to Chiari type I malformations, which has been attributed to these patients' narrow and shallow posterior fossa that crowd cranial nerves and vascular structures inside the cerebellopontine angle cistern [38, 39].

Collectively, these underlying issues of secondary HFS are thought to cause neural dysfunction and/or irritation of the facial nerve pathway [40]. Hearing loss, weakness of upper and low facial muscles, and preferential involvement of the orbicularis oculi and frontalis muscle were significantly more common in secondary HFS compared with primary HFS cases [7]. In a study of 252 patients, 78.5% presented with primary HFS whereas 21.5% presented with the secondary form [7]. Additional studies support that primary HFS is approximately 4 times more common than secondary HFS [30, 41].

## 5. Diagnosis 

The diagnosis of HFS is made clinically. The “Babinski-2 sign,” “other Babinski sign,” or “brow-lift sign” is a physical exam maneuver that is positive when a patient lifts his/her eyebrow with ipsilateral eye closure, signaling the synchronized activity of the frontalis and orbicularis oculi muscle during HFS [42–44]. This technique has been shown in one study to have high sensitivity (86%), specificity (100%), and interrater reliability (92%) for HFS diagnosis [45].

EMG, MRI, and computerized tomography (CT) are used to confirm the diagnosis and differentiate primary from secondary HFS. Of these modalities, T2-weighed MRI sequences and high resolution fast imaging employing thin section steady-state free precession MR images are most commonly used to display possible vascular compressions [21]. Fusion MR imaging that combines steady-state MR imaging and three-dimensional time-of-flight MR angiography has been shown to assist in describing patient-specific anatomy at the root exit zone of the facial nerve [46]. EMG can also be useful to differentiate HFS from other abnormal facial movement disorders; in HFS, spontaneous, high-frequency synchronized firing is seen on EMG [3]. Additional diagnostic techniques such as a CT angiogram are useful for microsurgical planning. A recent study also suggested that the hemodynamic changes may be detectable using color-duplex ultrasound, showing a higher mean blood flow velocity in PICA and AICA arteries on the HFS side compared to that of the contralateral face [47]. An analysis using three-dimensional MR volumetric analysis found that HFS patients have lower posterior fossa CSF volumes compared to that of matched controls, suggesting that smaller posterior fossa CSF space may be an HFS risk factor [48].

All these diagnostic techniques help differentiate HFS from other craniofacial dyskinesias such as blepharospasm (BSP), tic disorders, myokymia, and synkinesis in addition to other disorders such as partial motor seizures, craniocervical dystonia (Meige syndrome), tardive dyskinesias (TD) and neuromyotonia. Other conditions such as psychogenic HFS, facial myoclonus, oromandibular dystonia, and hemimasticatory spasm can masquerade as HFS, resulting in diagnostic difficulty [34]. One case of moyamoya disease presented as HFS and was identified due to facial nerve compression with compensatory posterior circulation vessel enlargement [49]. In addition, psychogenic HFS was found in 2.4% of patients evaluated for HFS in one study and can lead to unnecessary medical and/or surgical intervention [50].

Comorbidity between HFS and other craniofacial dyskinesias can occur. Trigeminal neuralgia (TN) is irritation of the trigeminal nerve that causes facial pain. It can present concurrently with HFS in a syndrome called tic convulsif. Studies have shown that HFS can follow Bell's palsy, which is facial paralysis from dysfunctional facial nerve caused by brain tumor, stroke, myasthenia gravis, and Lyme disease [34]. HFS has also been reported to occur as a result of facial nerve demyelination in multiple sclerosis patients.

## 6. Medical Treatment 

The standard medical treatment for HFS is botulinum neurotoxin (BoNT) injections. Having been used since the early 1980s, BoNT injections provide low-risk symptomatic relief in 85% of HFS patients, making it the treatment of choice for patients with high anesthetic risk and those who refuse surgery [21]. One study suggested that BONT-A also helped improve hemifacial spasm-related headaches [8].

BoNT's mechanism of action is to block calcium-mediated release of acetylcholine at the synaptic junction. Two serotypes are available: BoNT-A and BoNT-B, as well as four different commercial formulations: abobotulinumtoxinA, onabotulinumtoxinA, incobotulinumtoxinA, and rimabotulinumtoxinB [51]. After injection, BoNT is cleaved by trypsin into heavy and light chain components [52]. At this point, the BoNT toxin is internalized into presynaptic nerve terminals, where the heavy chain binds synaptic vesicle protein 2, trisialoganglioside 1b, and synaptotagmin-1 [53]. The light chain then binds to the SNARE complex and cleaves target proteins such as synaptosomal-associated proteins of 25 kDa (SNAP-25) and synpatobrevin-2 to prevent exocytosis of neurotransmitters from the presynaptic terminal, leading to muscle paralysis [54].

BoNT-A is the primary serotype used for HFS treatment. BoNT-A injections occur in several sites in the pretarsal and preseptal portions of the facial nerve and are effective with a mean onset of action of 3 to 5 days. In one longitudinal multicenter center study, the effectiveness of BoNT-A in relieving HFS symptoms remained unchanged in the first and tenth year with patients needing statistically similar doses [55]. However, the injections must be repeated every 3 to 6 months. Tolerance can develop in some cases, but the treatment is generally well tolerated. Local complications of these injections include ptosis, blurred vision, and diplopia that may improve after days to weeks [14]. Repeated injections also can cause atrophy of target muscles, which may lead to injection of the contralateral face for cosmetic reasons [54]. Despite the effectiveness and low complication rate of BoNT-A, the need for repeated injections incurs a high economic cost and provides only symptomatic relief. Comparatively, BoNT-B is less commonly used. In an open-label single dose study, BoNT-B serotype was also shown to be well tolerated with 40% of subjects responding to treatment [56].

Pharmaceuticals such as anticonvulsants and GABAergic drugs may be used as alternative to BoNT injections. These drugs are generally less effective compared to BoNT at treating HFS. No controlled studies have found demonstrated long-term effectiveness of these medications, limiting their treatment utility. However, they can be used for symptomatic relief in early HFS patients who have mild and infrequent symptoms as well as patients who decline BoNT injections and/or surgical intervention.

## 7. Surgical Treatment

As an alternative to BoNT injections, microvascular decompression (MVD) provides a curative treatment with long-term relief of symptoms by alleviating vascular compression of the facial nerve root. The underlying principle of MVD is to separate the nerve-vessel conflict rather than isolate it with prostheses; important intraoperative considerations include prompt identification of the neurovascular conflict site, sharp dissection of arachnoids for maximal nerve root visualization, and electrophysiological monitoring to distinguish offending vessels [57]. MVD has excellent results with long-term success rates between 83% and 97% of cases [58].

An analysis of twenty-two papers representing 5,685 patients treated with MVD for HFS found that an average of 91.1% of patients had complete resolution of symptoms over a median 2.9-year follow-up period [59]. Even with a first-time MVD failure, patients in one study who elected for repeated MVDs had a cure rate of 85% and did not suffer a higher rate of complication with a mean follow-up of 54.48 months [60]. Another small study found no significant difference between elderly and young patients in cure rate (96.3% versus 89.4%) and complication rate [61].

Before MVD, MRI imaging is used to identify the offending vessel and exclude structural pathology such as meningioma, acoustic neuromas, or epidermoid tumors. One study showed that preoperative assessment of HFS using T2-weighted MR cisternography predicted 79.1% of offending vessel invagination into the brainstem, allowing for better preoperative planning [62].

Under general anesthesia, the patient is typically placed in either supine or the lateral decubitus position [63]; a craniotomy inferior of the transverse sinus and medial of the sigmoid sinus is performed to expose the dura [14]. Once identified, the offending vessel can be mobilized and separated from the facial nerve root using shredded Teflon implants [64]. After the facial nerve is free of vascular contact, symptom resolution may occur immediately due to decreased compressive force [65]. Symptom resolution could be delayed, which is thought to be from remyelination at the microinjury site or normalization of the facial motor nucleus response [59, 66]. At the end of the MVD procedure, the dura is closed after irrigating the cerebellopontine angle and verifying that the Teflon implants are immobile. The senior authors (KRB/EM) replace the bone flap and perform a bone substitute cranioplasty [14].

Intraoperative EMG monitoring of facial nerve AMR increases safety of the operation and improves MVD outcomes. Outcomes of MVD can be optimized when the full length of the facial nerve is confirmed to be clear of the offending vessel, all offending vessels double-checked to be removed from the nerve, and AMRs disappear [67]. One study found that patients had a fourfold greater chance of HFS cure if AMR was abolished intraoperatively using EMG surveillance [3]. In the 38% of HFS patients with multiple neurovascular compression, AMR and ZL-Response (ZLR), an alternative intraoperative EMG, used simultaneously as intraoperative monitoring, has been suggested to provide more useful information than AMR alone especially in situations when AMR is unavailable or unstable; the study reported 92% HFS resolution rate in HFS patient with multiple neurovascular compressions using this method [68]. Monitoring lateral spread response (LSR) also correlates with MVD. Several studies show that the disappearance of LSR during decompression predicted favorable outcomes [69–71] whereas the disappearance of LSR during dural opening or after CSF drainage before decompression correlated with worse outcomes [72].

Individual surgical methods vary. One postoperative study with an average follow-up term of 13 years suggested a “supine, no retractor” system having fewer adverse effects during general anesthesia with lower risks of postoperative nausea/dizziness, peripheral nerve palsy, and deafness [73]. Techniques to preserve the lesser occipital nerve during the lateral suboccipital craniotomy portion of MVD have also been described and reduce the incidence of sensory disturbances in the occipital region [74]. Compressions of the facial nerve outside the root exit zone have been described and shown that entire-root-decompression technique provides improved outcomes compared to decompression of just the root exit zone [75]. Emphasis must also be placed on mobilizing offending arterioles in addition to larger arteries. One study of 69 patients with intraoperative EMG found that nine patient who had artery mobilization had persistent AMRs, which resolved after offending arterioles were also separated from the facial nerve [76]. In reexploratory surgeries, two factors that may have complicated the initial decompression include inadequate exposure of the root exit zone and the use of unshredded Teflon implants, which can be easily dislodged [64].

Resolution of HSF after MVD may take several months to several years with small percentage of patients who fail to improve. In these patients, failure to improve may be attributed to inadequate decompression of the offending vessel, presence of a previously unidentified secondary offending vessel, or implant compression/migration against the facial nerve [77]. Generally, complications of MVD are uncommon and generally transient [59]. In some cases, MVD can result in serious complications, which are thought to be caused by facial nerve stretching during cerebellar retraction, iatrogenic injury to surrounding structures, or prosthesis compression [78]. The most common are deafness (2% to 20% of cases) or partial hearing loss, defined by one study as pure tone audiometry of more than 10 dB at frequencies of 4 and 8 kHz (26.6%); follow-up and repeat audiological examination studies are still lacking [79]. The use of brainstem auditory evoked potential monitoring (BAEPs) during MVD may warn surgeons of cochlear nerve damage intraoperatively by following the latency of Wave 5, which corresponds to the brainstem auditory pathways from the cochlear nucleus to the inferior colliculus [78].

A few cases of cerebrospinal fluid (CSF) leakage, cerebellar injury, and lower cranial nerve complications have been reported as well as life-threatening complications, such as space-occupying hemorrhages and cerebellar/brainstem infarctions [61]. A retrospective comparison study suggested that CSF leakage occurs with postoperative use of closed-suction drainage [80]. Calcium phosphate cement following retromastoid craniectomies has been suggested to decrease the rate of complications such as CSF leaks with satisfactory cosmetic outcomes [81]. Overall, serious complications following MVD were reported in less than 1% of cases [21]. HFS recurrence can occur in 4% to 10% of patients [21] and has been associated with arterial hypertension [82]. MVD can also be used to treat the rare patients with coexistent HFS, trigeminal neuralgia, and glossopharyngeal neuralgia [83] as well as patients with cerebellopontine angle tumors when combined with tumor removal [84].

Hospital-wide protocols are also optimizing patient outcomes and containing costs. After one institution implemented enhanced-recovery perioperative protocols and diagnosis-specific clinical pathways in patients undergoing MVD for HFS and trigeminal neuralgia, it reported decreased operating room times, hospital stay length, and a reduction in rates of complications and readmissions [85]. Concurrently, a retrospective study at the same institution reduced surgical care episodes costs by 25% by decreasing duration of operations and simplifying intraoperative monitoring intraoperatively while reducing ICU and total hospital stay length postoperatively [86].

Overall, MVD remains the treatment of choice for patients with HFS as the development of intraoperative technique and monitoring continues to improve.
